# Prognostic significance of metastatic lymph node ratio: the lymph node ratio could be a prognostic indicator for patients with gastric cancer

**DOI:** 10.1186/s12957-018-1504-5

**Published:** 2018-10-04

**Authors:** Yi Hou, Xudong Wang, Jing Chen

**Affiliations:** Department of Gastrointestinal Surgery, The Fourth Affiliated Hospital of China Medical University, Chongshan road 4th, Huanggu district, Shenyang, 110032 Liaoning China

**Keywords:** Gastric cancer, Lymph node ratio, Survival analysis, Multivariate analysis, Prognosis

## Abstract

**Background:**

To demonstrate the prognostic significance and value of lymph node ratio (LNR) and evaluate the possibility of becoming a new indicator to enhance the current Union for International Cancer Control (UICC)/American Joint Committee on Cancer (AJCC) tumor, lymph node, metastasis (TNM) staging system.

**Methods:**

Our retrospective study included 221 patients who got gastric cancer and underwent curative gastrectomy between 2005 and 2012 at the Fourth Hospital Affiliated of China Medical University. The log-rank test was used to compare the clinicopathological variables. The Kaplan-Meier method and Cox proportional hazard regression model was used to perform the univariate analysis and multivariate statistical survival analysis.

**Results:**

The patients with a better differentiated pathological type; an earlier stage of T staging, N staging, and TNM staging; and a lesser LNR would have a longer survival time according to the univariate analysis. As for the multivariate analysis, the Grade, T stage, N stage, and LNR had the statistical significance. Both in group 1 (the number of lymph nodes examined ≥ 15, namely LN ≥ 15) and group 2 (LN < 15), the LNR had statistical significance and the median survival time would decrease with the increase of the LNR. It was still statistically significant between group LNR1 and group LNR2 which were regrouped by the new cut-off value.

**Conclusion:**

The LNR could estimate the prognosis of patients with curative gastrectomy regardless of the number of lymph nodes examined. Thus LNR could become a new indicator to enhance the current TNM stage system.

## Background

The study aimed to demonstrate the prognostic significance and value of lymph node ratio (LNR) and evaluate the possibility of becoming a new indicator to enhance the current Union for International Cancer Control (UICC)/American Joint Committee on Cancer (AJCC) tumor, lymph node, metastasis (TNM) staging system.

## Main text

### Introduction

Gastric cancer is one of the most common malignancies and was one of the five most commonly diagnosed cancers in China in 2015. The estimated incidence in 2015 was 679,100, including 477,700 men and 201,400 women. It was also the second leading cause of cancer death in China, with an estimated total mortality of 498,000, including 339,300 men and 158,700 women. Worldwide, gastric cancer was also the fourth most common cancer. [1, 2]. Thereby, adequate and timely treatment is necessary for patients with gastric cancer. Curative resection remains the most essential treatment for patients with gastric cancer. However, postoperative clinical pathological staging is equally crucial for guiding postoperative therapy. The most commonly and extensively used staging system for gastric cancer is the Union for International Cancer Control (UICC)/American Joint Committee on Cancer (AJCC) tumor, lymph node, and metastases (TNM) staging system. The TNM staging system classifies patients with gastric cancer into various stages based on the depth of primary tumor invasion (T stage), regional lymph node metastases (N stage), and distant metastases (M stage) [3–5]. However, “stage migration” is frequent and occurs in 10–25% of cases [6]. The 7th edition TNM staging system requires that at least 15 lymph nodes be examined to obtain an accurate lymph node metastatic category. However, the surgeon’s technical expertise, the pathologist’s experience, and other unavoidable conditions may result in less than 15 lymph nodes examined, which has been deemed inadequate [7].

The phenomenon of stage migration is caused by an insufficient number of lymph nodes examined [4, 5, 8]. This phenomenon can lead to inaccurate classification and may affect guidance for postoperative therapy. In order to reduce stage migration, some investigators have proposed using the LNR, namely the ratio between positive lymph nodes compared with the total number of lymph nodes examined, as a new prognostic indicator for gastric cancer. LNR has been confirmed to be a simple and reproducible prognostic tool, even in the case of limited lymph node dissection [6]. There have been a series of reports that show that LNR may effectively reduce the phenomenon of stage migration. Additionally, some studies have reported LNR to be an independent prognostic factor [8–13].

In the present study, we retrospectively evaluated the prognostic significance of LNR in 221 gastric cancer patients. We aimed to evaluate the prognostic significance and clinical value of the metastatic LNR in patients who underwent curative gastrectomy, with a potential goal of enhancing and the 7th edition TNM staging system.

## Methods

### Patients

This retrospective study included 221 patients who underwent curative gastrectomy for a definite histological diagnosis of gastric cancer between 2005 and 2012 at the Fourth Hospital Affiliated of China Medical University. All 221 candidates had undergone chest radiography, abdominal computed tomography (CT), and gastroscopy. Patient eligibility criteria included the following: [1] R0 curative gastrectomy, [2] accurate histopathological examination, [3] no less than a D2 lymph node dissection, [4] no identifiable distant metastasis in the liver, peritoneum, and so on, [5] no recurrent gastric carcinoma or gastric stump carcinoma, [7] survived the perioperative period, [8] no neoadjuvant chemotherapy or other preoperative chemotherapy, and [9] complete medical record and follow-up data.

R0 curative gastrectomy was defined as no macroscopic and microscopic remaining tumor tissue in the margin of the resected specimens. D2 lymphadenectomy involved the removal of the N1 nodes, defined as the perigastric lymph node stations 1, 3, and 5 along the lesser curvature of the stomach and perigastric lymph node stations 2, 4, and 6 along the greater curvature of the stomach. N2 was defined as perigastric lymph node stations 7 (along the left gastric artery), 8 (along the common hepatic artery), 9 (along the celiac artery), and 10 (along the splenic artery) [14].

Study patients were divided to two groups. Group 1 included 178 patients who had 15 or more lymph nodes examined (sufficient group). Group 2 included 43 patients who had less than 15 lymph nodes examined (insufficient group).

Our study was performed in accordance with the ethical standards of the World Medical Association Declaration of Helsinki. All 221 patients provided their written informed consent to participate in this study. Our study was approved by the independent ethics committees at the Fourth Hospital Affiliated of China Medical University.

### Statistical analysis

SPSS (Statistical Product and Service Solutions) software version 19.0 for Windows (SPSS Inc. Chicago, IL, USA) was used for all statistical analyses. The differences between clinicopathological variables were compared by the Kaplan-Meier method. The statistical significance of the differences between different survival curves was examined by the log-rank test. The Cox proportional hazard regression model was used to perform multivariate statistical survival analysis. The cut-off values of subgroups of T stage, N stage, and TNM stage were based on the 7th AJCC/UICC TNM staging system. The cut-off values of LNR were 0, 0.13 (2/15), and 0.4 (6/15). The subgroups of LNR were defined as R0 (LNR = 0), R1 (0 < LNR ≤ 0.13), R2 (0.13 < LNR ≤ 0.4), and R3 (LNR > 0.4). The independent variables analyzed were as follows: [1] sex (male versus female), [2] age (< 65 versus ≥ 65), [3] tumor location (lower third versus middle third versus upper third), [4] grade (poorly differentiated versus well differentiated and moderately differentiated), [5] T stage (T1 versus T2 versus T3 versus T4), [7] N stage (N0 versus N1 versus N2 versus N3), [8] TNM stage (I versus II versus III), [9] total number of examined lymph nodes (< 15 versus ≥15), and [10] the ratio between metastatic lymph nodes and examined lymph nodes (R0 versus R1 versus R2 versus R3). A *p* value of less than 0.05 was defined as statistically significant for all analyses in this study.

## Results

### Clinical and histopathology data

Of 221 patients, 160 (72.4%) patients were male and 61 (27.4%) were female. The median age was 64 (range 37 to 85) years. The median survival time was 42 months with a 5-year survival rate of 29.0%. There was a total of 6606 lymph nodes resected with an average of 29.9 ± 1.1 (mean ± standard error) and a median of 30 (range 1 to 105). The number of patients who had greater than or equal to 15 lymph nodes resected was 178 (80.5%); 43 patients (19.5%) had fewer than 15 lymph nodes resected. There were 1503 positive lymph nodes in the entire cohort, with an average of 6.8 ± 0.7 (mean ± standard error) and a median of 2 (range 0 to 50). Regarding tumor grade, 51 (23.1%) patients had tumors that were well differentiated or moderately differentiated histologically; 170 (76.9%) had poorly differentiated tumors. Regarding tumor location, there were 180 (81.4%), 13 (5.9%), and 28 (12.7%) tumors in the lower, middle, and upper groups, respectively. Patients were divided to four groups based on T stage (T1, T2, T3, and T4); there were 23 (10.4%), 39 (17.6%), 98 (44.3%), and 61 (27.6%) patients in each group respectively. Concerning N stage, there were 67 (30.3%), 46 (20.8%), 34 (15.4%), and 74 (33.5%) patients in the N0, N1, N2, and N3 groups, respectively. With regard to TNM stage, all patients were divided into three groups according to stage I, II, and III; there were 43 (19.5%), 63 (28.5%), and 115 (52.0%) patients in each staging group, respectively. All clinical and histopathology data is presented in Table 1.

**Table 1:** Clinical and histopathology data of all 221 patients

Variables	Number of patients	Percent (%)
Sex		
Male	160	72.4
Female	61	27.6
Age (years)		
≥ 65	100	45.2
< 65	121	54.8
Grade		
Well or moderately differentiated	51	23.1
Poorly differentiated	170	76.9
Location		
Lower	180	81.4
Middle	13	5.9
Upper	28	12.7
T stage		
T1	23	10.4
T2	39	17.6
T3	98	44.3
T4	61	27.6
N stage		
N0	67	30.3
N1	46	20.8
N2	34	15.4
N3	74	33.5
TNM stage		
I	43	19.5
II	63	28.5
III	115	52.0
LN		
< 15	43	19.5
≥ 15	178	80.5
LNR		
0	68	30.8
0–0.13	47	21.3
0.13–0.4	54	24.4
> 0.4	52	23.5

The depth of primary tumor invasion (T stage), classification of regional metastasis lymph nodes (N stage), and TNM stage were based on the 7th edition TNM staging system; LN: number of lymph nodes examined; LNR: ratio between the positive lymph nodes and the total number of lymph nodes examined

### Univariate and multivariate analysis

**Fig. 1:**
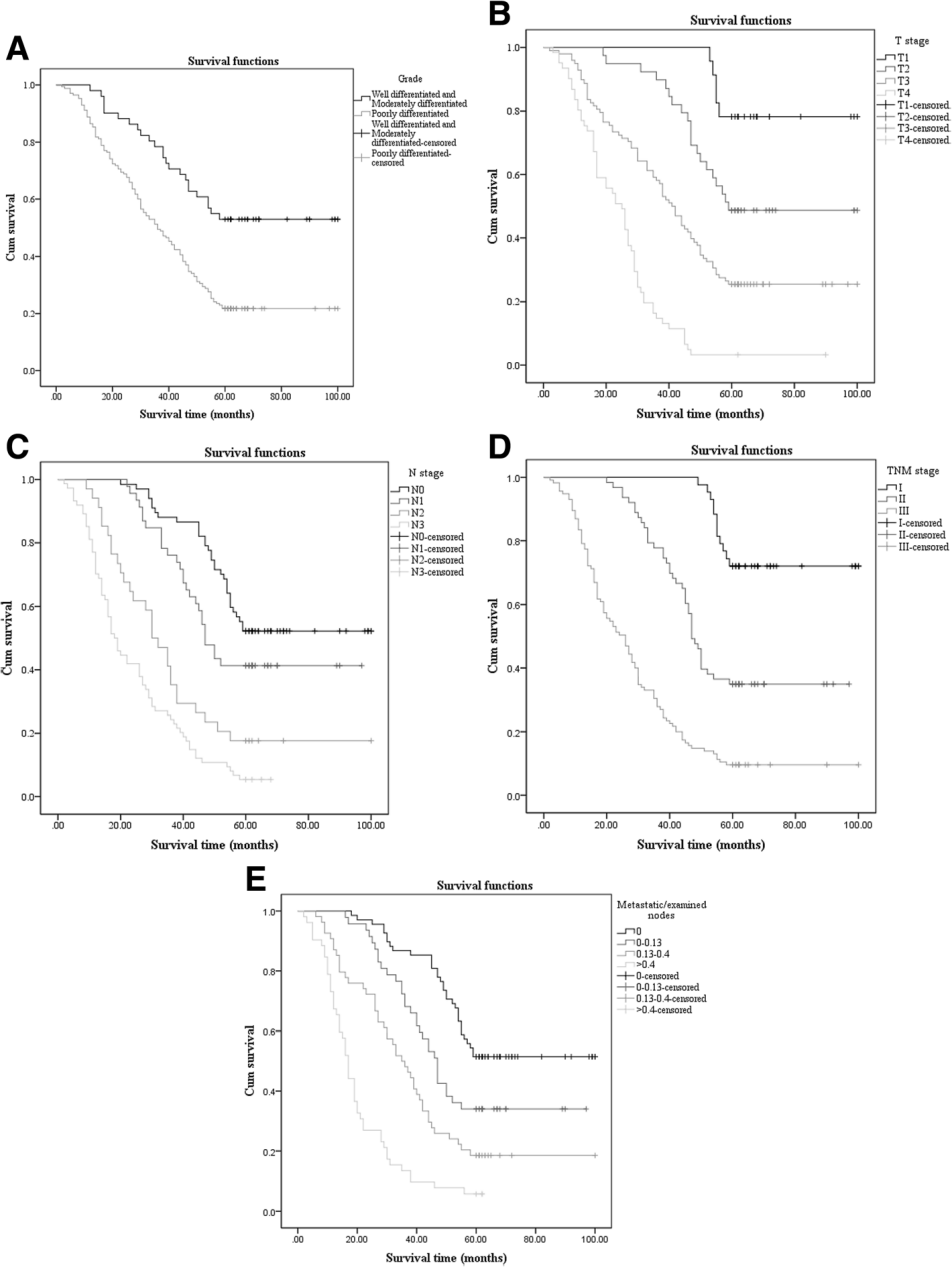
The factors with statistical significance of univariate analysis and panels **a** to **e** reveal the survival curves of grade, T stage, N stage, TNM stage, and LNR, respectively

In the univariate analysis, there were nine clinicopathological variables tested to verify statistical significance in comparing overall survival (OS) among all 221 patients. The clinicopathological variables included sex, age at surgery, tumor grade, tumor location, T stage, N stage, TNM stage, LN (the number of lymph nodes resected), and LNR (the ratio between metastatic lymph nodes and examined lymph nodes). Ultimately, tumor grade (*p* < 0.001), T stage (*p* < 0.001), N stage (*p* < 0.001), TNM stage (*p* < 0.001), and LNR (*p* < 0.001) were statistically significant (Fig. 1). The results of the univariate analysis, which included median survival time and *p* value, are presented in Table 2. All nine clinicopathological variables were included in the multivariate analysis by the Cox proportional-hazards model (forward stepwise procedure). The multivariate analysis showed that tumor grade, T stage, N stage, and LNR still had statistical significance. The result of the multivariate analysis is presented in Table 3.

**Table 2:** Univariate Analysis of 221 Patients with curative gastrectomy

Variables	Median survival (month)	*p* value
Sex		0.824
Male	44.0	
Female	41.0	
Age (years)		0.482
≥ 65	41.0	
< 65	42.0	
Grade		< 0.001
Well or moderately differentiated	61.0	
Poorly differentiated	36.0	
Location		0.405
Lower	40.5	
Middle	45.0	
Upper	50.0	
T stage		< 0.001
T1	64.0	
T2	59.0	
T3	41.5	
T4	25.0	
N stage		< 0.001
N0	60.0	
N1	47.0	
N2	31.0	
N3	18.5	
TNM stage		< 0.001
I	62.0	
II	47.0	
III	26.0	
LN		0.895
< 15	44.0	
≥ 15	41.5	
LNR		< 0.001
0	60.0	
0–0.13	47.0	
0.13–0.4	35.5	
> 0.4	17.0	

The depth of primary tumor invasion (T stage), classification of regional metastasis lymph nodes (N stage) and TNM stage were based on the 7th edition TNM staging system; LN: number of lymph nodes examined; LNR: ratio between the positive lymph nodes and the total number of lymph nodes examined

**Table 3:** Multivariable analysis of all Variables using Cox proportional hazard regression model

Variables	HR	*p* value	95.0% CI	
			Lower	Upper
Grade				
Well or moderately differentiated	1			
Poorly differentiated	0.442	< 0.001	0.284	0.689
T stage				
T1	1			
T2	0.086	< 0.001	0.033	0.228
T3	0.225	< 0.001	0.128	0.395
T4	0.277	< 0.001	0.189	0.407
N stage				
N0	1			
N1	0.066	0.009	0.009	0.507
N2	0.341	0.002	0.172	0.674
N3	0.580	0.036	0.348	0.965
LNR				
0	1			
0–0.13	2.358	0.402	0.317	17.519
0.13–0.4	0.721	0.342	0.368	1.414
> 0.4	0.427	< 0.001	0.277	0.659

*CI*: confidence interval

**Fig. 2:**
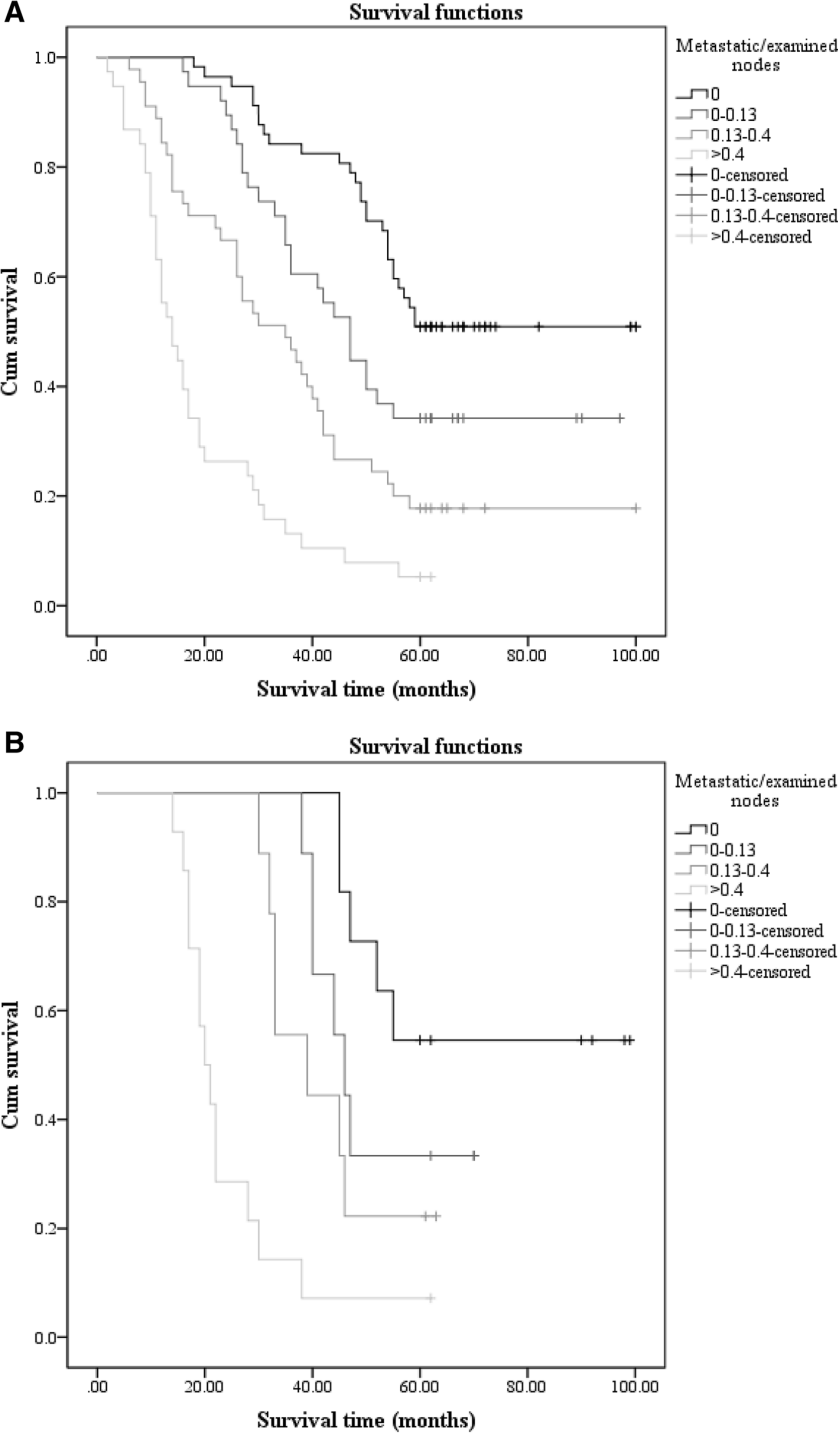
The survival curves of LNR in group 1 (LN ≥ 15) and group 2 (LN < 15), respectively. Panels **a** and **b** reveal the survival curves of LNR in group 1 (LN ≥ 15) and LNR in group 2 (LN < 15), respectively

In this study, we focused on LNR (the ratio between metastatic lymph nodes and examined lymph nodes) in the sufficient group (group 1, LN ≥ 15) and the insufficient group (group 2, LN < 15). In group 1, the total number of patients who had greater than or equal to 15 lymph nodes resected was 178; there were 57 (32.0%), 38 (21.3%), 45 (25.3%), and 38 (21.3%) patients in the r0 (LNR = 0), r1 (0 < LNR ≤ 0.13), r2 (0.13 < LNR ≤ 0.4), and r3 (LNR > 0.4) groups, respectively. The univariate analysis showed a statistically significant result (*p* < 0.001) in comparing LNR (Fig. 2a). On the other hand, there were 43 patients who had fewer than 15 lymph nodes in group 2. This group had the following resection rates: r0 (LNR = 0), r1 (0 < LNR ≤ 0.13), r2 (0.13 < LNR ≤ 0.4), and r3 (LNR > 0.4) had 11 (25.6%), 9 (20.9%), 9 (20.9%), and 14 (32.6%) patients, respectively. We still obtained a statistically significant result (*p* < 0.001) in the univariate analysis (Fig. 2b). The results of the univariate analysis of LNR in groups 1 and 2 are presented in Table 4.

**Table 4:** Univariate analysis of LNR in groups with ≥ 15 and < 15 lymph nodes examined

	Number of patients	Percent (%)	Median survival (month)	*p* value
Group 1				
LNR				< 0.001
0	57	32.0	60.0	
0–0.13	38	21.3	47.0	
0.13–0.4	45	25.3	35.0	
> 0.4	38	21.3	14.0	
Group 2				
LNR				< 0.001
0	11	25.6	60.0	
0–0.13	9	20.9	46.0	
0.13–0.4	9	20.9	39.0	
> 0.4	14	32.6	20.5	

**Fig. 3:**
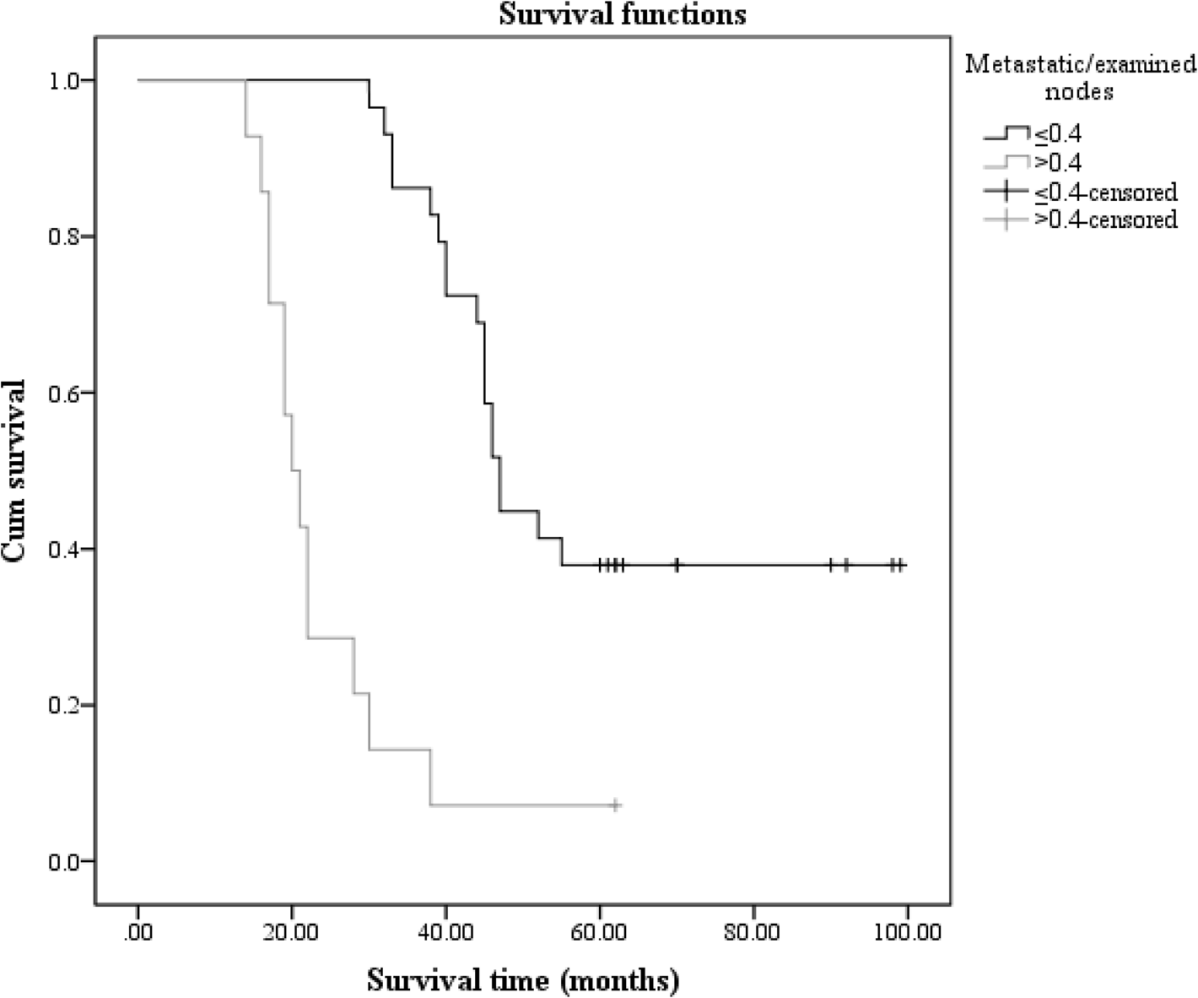
The survival curves of LNR in group 2 (LN < 15) with the new cut-off

Although the univariate analysis showed a statistically significant result in group 2, we abandoned the method that divided patients in group 2 into four subgroups. We found another way to evaluate prognostic significance of LNR in group 2, to make the result more accurate. We compared all 221 patients who had R0 resections with those who had R1, R2, and R3 resections, respectively. Then, we found that R3 had the maximum chi-square value. These results are shown in Table 5. Finally, we chose 0.4 as a new cut-off value in group 2 and divided patients into two groups, LNr1 (LN ≤ 0.4) and LNr2 (LN > 0.4). We were then able to obtain a statistically significant result (*p* value < 0.001) by comparing these two subgroups (Fig. 3). The LNr1 and LNr2 groups had 29 (67.4%) and 14 (32.6%) patients, respectively.

**Table 5:** Comparisons of overall survival between R0 and R1, R2, or R3

	Median survival (month)	Chi-square	*p* value
R1	47.0	6.999	0.008
R2	35.5	28.101	< 0.001
R3	17.0	82.490	< 0.001

## Discussion

Gastric cancer, one of the most common malignant neoplasms in the world, results in the death of thousands every year, especially in China [1, 5, 15]. After curative resection of gastric cancer was implemented, the possibility to extend survival has been a topic of exploration for investigators globally, as extending life is always a consistent goal. Thus, the factors that influence prognosis after curative resection in gastric cancer have been extensively studied. Indisputably, lymph node stage (N stage) is one of the foremost prognostic factors [16–18]. Many studies have shown that the 5-year survival rate of gastric cancer patients with positive lymph nodes is significantly lower than in those without lymph node metastasis. Moreover, as the number of lymph node metastases increases, prognosis gradually decreases. Not only metastasis lymph node stage but also the total number of lymph nodes examined is an important factor that influences prognosis. It has been demonstrated that the number of lymph nodes is an independent prognostic factor and a larger number of lymph nodes can lead to a higher 5-year overall survival rate [19–24]. The TNM staging system, a tool to evaluate prognosis of patients who had curative resection of gastric cancer, is current and accepted comprehensively by surgeons. In the 7th Union for International Cancer Control (UICC)/American Joint Committee on Cancer (AJCC) tumor, lymph node, metastasis (TNM) staging system published in 2010, metastatic lymph nodes are essential in prognostication. However, properly classifying lymph node metastasis is limited by the number of lymph nodes. This system requires that at least 15 lymph nodes be examined postoperatively to obtain precise N staging, in order to avoid inaccurate staging. When the number of lymph nodes is > 15, the number of lymph node metastases is more accurate in assessing prognosis. However, if the number of lymph nodes is insufficient, the phenomenon of stage migration occurs [4, 5, 8, 10]. In addition, increasing the number of lymph nodes examined can lead to a higher 5-year survival rate. Hence, obtaining more lymph nodes from the postoperative specimen was deemed to be necessary and useful. Most surgeons follow the UICC/AJCC guide and remove a sufficient number of lymph nodes. Nevertheless, there are still some reasons that lead to fewer than 15 lymph nodes being obtained at surgery. Insufficiency of the technique itself, surgeon experience, or the lymph nodes in the specimen being too small may be reasons leading to a lesser number of lymph nodes being examined [7, 25]. Thus, many investigators have investigated finding a method to reduce that phenomenon. In recent years, LNR has been provided superior prognostic information over the N category according to the TNM classification in breast, colon, and rectal cancer [26]. Some investigators have proposed that LNR could be a new prognostic indicator and have demonstrated LNR to be an independent prognostic factor in gastric cancer. It has also been attested that the LNR may reduce the phenomenon of stage migration [10, 13, 27–29].

We aimed to determine the prognostic significance of the metastatic LNR as a new tool to evaluate prognosis of patients with curative gastrectomy. In our study, we found that tumor grade, T stage, N stage, TNM stage, and LNR were the factors that influenced prognosis of patients according to the univariate analysis. Patients with a better differentiated pathological type, an earlier stage of T staging, N staging, and TNM staging, and a lower LNR have improved survival rates. However, when all nine factors are entered into the Cox proportional-hazards model, the multivariable analysis showed that only grade, T stage, N stage, and LNR showed statistical significance. LNR still had statistical significance in both the univariate and multivariable analysis. Thus, our study again demonstrated that LNR was an independent prognostic factor. With increased LNR, OS decreases. Thus, LNR may have value for evaluating prognosis. LNR could become a new tool to estimate prognosis in patients who undergo curative gastrectomy.

Although LNR is an independent prognostic factor, further research is required. We have evaluated the influence of LNR on prognosis in group 1 (LN ≥ 15) and group 2 (LN < 15). In our study, we set cut-off values (0, 0.13, and 0.4) based on N stage of the TNM staging system. The advantages of and reasons for choosing this cut-off value were convenience and ease, which should be important characteristics for any prognostic system used by physicians. Ultimately, we divided all patients in each group into four subgroups (R0, R1, R2, and R3) according to LNR, respectively.

In group 1, there were 178 patients, who were divided into the following four subgroups: r0 (LNR = 0), r1 (0 < LNR ≤ 0.13), r2 (0.13 < LNR ≤ 0.4), and r3 (LNR > 0.4). We compared the four subgroups with regard to survival time, and the univariate analysis showed statistical significance between the four subgroups. Patients in the r0 group had a maximal median survival time of 60.0 months, and the median survival time of patients in the r3 group was minimal (14.0 months). Thus, we considered that when LN ≥ 15, the LNR had value in evaluating prognosis of patients with curative gastrectomy and the median survival time decreased with increasing LNR. In group 2, we still obtained a statistically significant result between r0 (LNR = 0), r1 (0 < LNR ≤ 0.13), r2 (0.13 < LNR ≤ 0.4), and r3 (LNR > 0.4). The univariate analysis showed that different LNRs can lead to different prognoses.

It appeared that LNR may be a prognostic indicator for patients, regardless of number of lymph nodes examined, according to our study results. However, we did not think that the method of grouping that divided all patients into four groups was suitable for group 2. On the one hand, we had a small sample size and the number of patients with LN < 15 examined was only 43. On the other hand, when the number of lymph nodes examined was less than 15, increasing or decreasing the number by one lymph node would lead to a larger variation of LNR. For example, when the number of metastatic lymph nodes increased by one for patients with five lymph nodes examined, the LNR would increase by 0.2. But the LNR would increase by only 0.07 when the total number of lymph nodes examined was 15. Thus, it would be imprecise for prognostication if we divided the patients with fewer than 15 lymph nodes into too many subgroups.

Finally, we decided to divide our patients into two subgroups: LNr1 and LNr2. The cut-off value was chosen in this way: we compared all four subgroups of LNR, regardless of the number of lymph nodes examined. We compared R0 with R1, R2, and R3 and found that R3 had the largest significant statistical difference compared with R0. Ultimately, we chose 0.4 as the cut-off value and divided patients with LN < 15 examined into two subgroups. The univariate analysis showed a statistically significant result (Fig. 3). And the median survival time of patients with LNR that greater than 0.4 was 20.5 months. The other patients who had an LNR less than or equal to 0.4 had a higher median survival time (47.0 months). That result meant that LNR had value in evaluating prognosis of patients with fewer than 15 lymph nodes examined and the median survival time decreased with increasing LNR.

The TNM staging system has some disadvantages that could be improved. LNR, as a new research direction, has been shown to have value in estimating prognosis. Our study demonstrated that LNR was an independent prognostic factor. Either in patients with greater than or equal to 15 LN examined, or fewer than 15 LN, LNR could estimate prognosis and OS was shown to decrease with increasing LNR. We found that there was no correlation between LNR and the total number of harvested LNs. In other words, patients with identical LNR, even with differing numbers of detected metastatic nodes, will have a similar outcome. Conversely, among patients with the same number of metastatic nodes, those with a higher LNR will have an unfavorable outcome [30]. Thus, the LNR could be a new prognostic indicator to enhance the TNM staging system.

## Conclusions

In conclusion, LNR can estimate prognosis in patients who undergo curative gastrectomy, regardless of the number of LNs examined. Thus, LNR may become a new indicator to evaluate prognosis after curative gastrectomy and enhance the current TNM staging system.

## Abbreviations

AJCC: American Joint Committee on Cancer
CT: Computed tomography
LNR: Lymph node ratio
OS: Overall survival
SPSS: Statistical Product and Service Solutions
TNM: Tumor, lymph node, metastasis
UICC: Union for International Cancer Control
